# Approaches towards the synthesis of 5-aminopyrazoles

**DOI:** 10.3762/bjoc.7.25

**Published:** 2011-02-09

**Authors:** Ranjana Aggarwal, Vinod Kumar, Rajiv Kumar, Shiv P Singh

**Affiliations:** 1 Department of Chemistry, Kurukshetra University, Kurukshetra-136 119, Haryana, India; 2 Department of Chemistry, Maharishi Markandeshwar University, Mullana, Ambala-133 203, Haryana, India

**Keywords:** alkylidenemalononitriles, 5-aminopyrazoles, hydrazines, β-ketonitriles, malononitriles

## Abstract

The biological and medicinal properties of 5-aminopyrazoles have prompted enormous research aimed at developing synthetic routes to these heterocyles. This review focuses on the biological properties associated with this system. Various synthetic methods developed up to 2010 for these compounds are described, particularly those that involve the reactions of β-ketonitriles, malononitrile, alkylidenemalononitriles and their derivatives with hydrazines, as well as some novel miscellaneous methods.

## Review

The 5-aminopyrazole system represents an important heterocyclic template that has attracted considerable interest because of its long history of application in the pharmaceutical and agrochemical industries [1–4]. These compounds have been extensively investigated over the past one hundred years and their chemistry has been reviewed in two books published in 1964 [5] and in 1967 [6].

**Figure 1:**
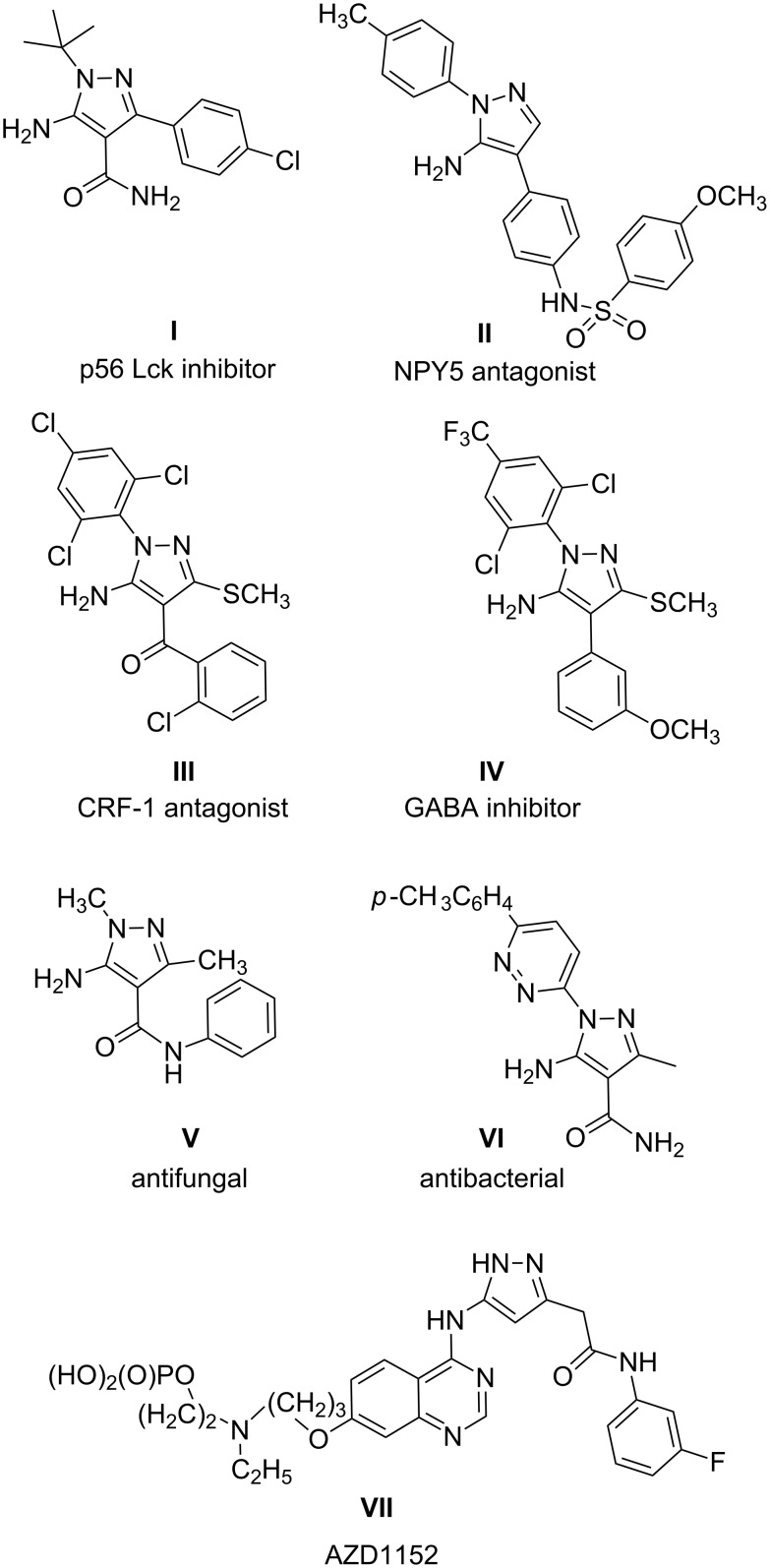
Pharmacologically active 5-aminopyrazoles.

Structurally simple 5-amino-1-*tert*-butylpyrazole-4-carboxamide **I** was found to inhibit p56 Lck [7] (Figure 1). 5-Amino-1-(4-methylphenyl) pyrazole **II** has been tested as an NPY5 antagonist [8]. 5-Amino-4-benzoyl-3-methylthio-1-(2,4,6-trichlorophenyl)pyrazole **III** has been reported as a potent corticotrophin-releasing factor-1 (CRF-1) receptor antagonist [9]. 5-Amino-1-(2,6-dichloro-4-(trifluoromethyl)phenyl)-4-(3-methoxyphenyl)-3-methylthiopyrazole **IV** has been described as a potent GABA inhibitor with selectivity towards insect versus mammalian receptors [10]. The simple *N*-phenyl amide of 5-amino -1,3-dimethylpyrazole-4-carboxylic acid **V** has been shown to exhibit antifungal activity [11] (Figure 1). The 5-amino-1-pyrazinyl-3-carboxamidopyrazole derivative **VI** has been recently reported as a potent antibacterial agent with a very broad spectrum [12]. Recently, components of the mitotic machinery have been targeted in an attempt to develop novel anticancer agents. These include critical signaling kinases such as the Aurora, PLK, and the cyclin-dependent kinases (CDK). Compound **VII** (AZD1152) is the first Aurora-B selective inhibitor to enter clinical trials [13] (Figure 1).

Besides the importance of 5-aminopyrazoles as biologically active agents, they are also useful synthons and building blocks for many heterocyclic products and can act as a binucleophile [14–18]. Cyclocondensation of 5-aminopyrazoles with 1,3-dielectrophiles has been extensively used for the preparation of bicyclic nitrogen heterocycles, especially in the preparation of condensed heterocycles such as pyrazolo[3,4-*d*]pyrimidines, pyrazolo[3,4-*b*]pyridines, imidazopyrazoles etc.

In view of significant interest in the synthesis of these heterocyclics, we herein report a detailed account of the synthetic methods available for 5-aminopyrazoles.

As pyrazole derivatives do not exist in nature, probably, due to the difficulty in the construction of N–N bond by living organisms, their availability depends on the synthetic methods. A large number of synthetic methods have recently appeared. Some of the important methods are outlined below.

### 1. Reaction of β-ketonitriles with hydrazines

**Scheme 1:**
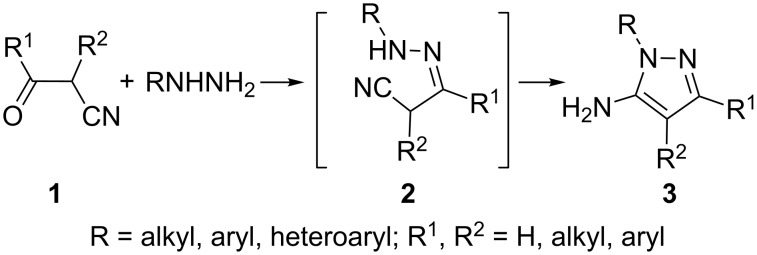
General equation for the condensation of β-ketonitriles with hydrazines.

The most versatile method available for the synthesis of 5-aminopyrazoles involves the condensation of β-ketonitriles with hydrazines. β-Ketonitriles **1** react smoothly with hydrazines to yield 5-aminopyrazoles **3** [19–28]. The reaction apparently involves the nucleophilic attack of the terminal nitrogen of the hydrazine on the carbonyl carbon with the formation of hydrazones **2**, which subsequently undergo cyclization by the attack of the other nitrogen on the nitrile carbon to produce 5-aminopyrazoles **3** (Scheme 1). Utilizing this reaction, a large number of 5-amino-1-heteroarylpyrazoles have been synthesized in our laboratory by the reaction of several heteroarylhydrazines with α-cyanoacetophenones [29–30]. The intermediate hydrazones **2** are rarely isolated, though their formation has been reported in the reaction of 2-nitro/2,4-dinitrophenylhydrazines and aryl-α-cyanoacetaldehydes **1** (R^2^ = aryl, R^1^ = H) [31].

**Scheme 2:**
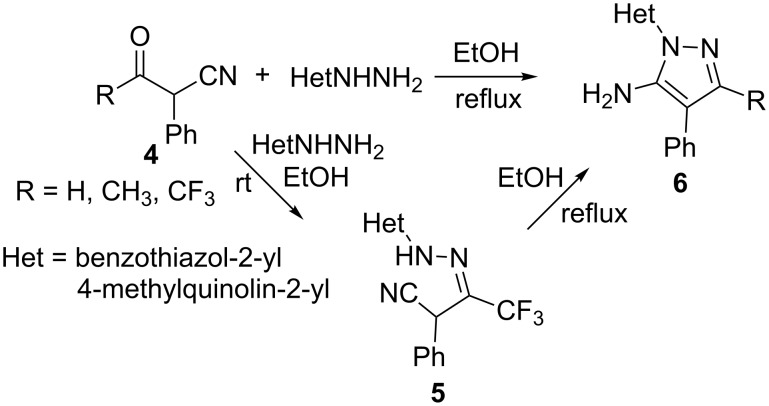
Reaction of hydrazinoheterocycles with α-phenyl-β-cyanoketones (**4**).

Recently, the synthesis of biologically active 5-amino-1-heteroaryl-3-trifluoromethylpyrazoles **6** has been achieved by us by the reaction of trifluoroacetylbenzyl cyanide **4** with heteroarylhydrazines [32]. The reaction of 2-hydrazino-4-methylquinoline with α-trifluoroacetylbenzyl cyanide (R = CF_3_) (**4**) at room temperature afforded the intermediate hydrazone **5**. The hydrazone **5** was characterized by IR and NMR spectroscopy. The IR spectrum of **5** showed a fundamental stretching band due to C≡N at 2179 cm^−1^. The ^19^F NMR spectrum of compound **5** showed fluorine signal at δ −65 ppm due to CF_3_ group confirming the formation of hydrazone **5**, which exists as the *Z*-isomer. From the literature [33], the signal for the CF_3_ group in trifluoromethylhydrazones appears at δ −64 to −66 ppm for *Z*-isomers and at δ −67 to −71 ppm for *E*-isomers. As expected, **5** underwent cyclization in refluxing ethanol to give the corresponding 5-aminopyrazole **6** [32]. α-Acetyl/formylbenzyl cyanide (R = H/CH_3_) **4** on reaction with heteroarylhydrazines in refluxing ethanol yielded the corresponding 5-amino-4-phenylpyrazoles **6**. These compounds were found to be good antibacterial agents (Scheme 2) [34].

**Scheme 3:**
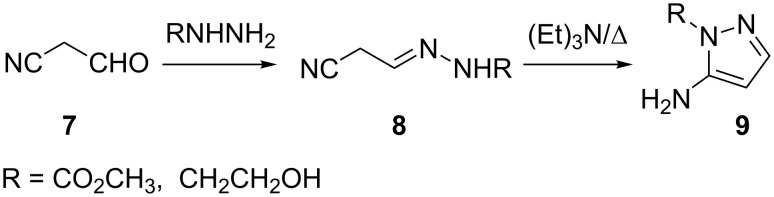
Condensation of cyanoacetaldehyde (**7**) with hydrazines.

The isolation of hydrazones **8** has also been reported during the condensation of cyanoacetaldehyde (**7**) with hydrazines [35]. These hydrazones **8** were cyclized to the corresponding 5-aminopyrazoles **9** under basic conditions (Scheme 3).

**Scheme 4:**
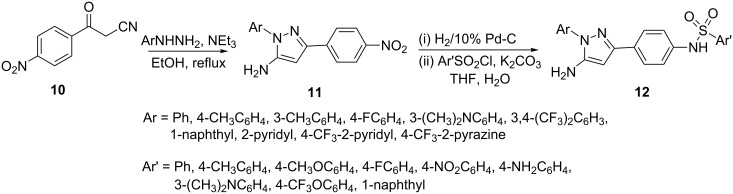
Synthesis of 5-aminopyrazoles and their sulfonamide derivatives.

Recently, Kordik et al [36] treated α-cyano-4-nitroacetophenone (**10**) with aryl hydrazines in the presence of triethylamine and obtained the corresponding 5-aminopyrazoles **11** in excellent yields. The latter were further converted into their sulfonamide derivatives **12** by reducing the nitro group to an amino group by catalytic hydrogenation followed by treatment with an arylsulfonyl chloride (Scheme 4).

**Scheme 5:**
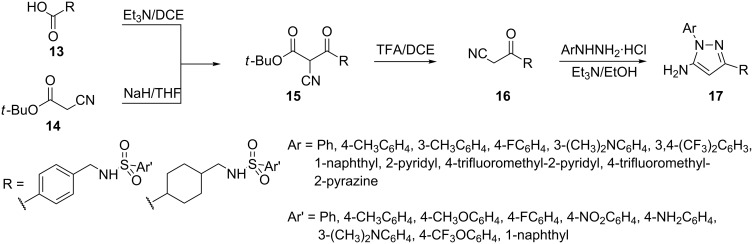
Synthesis of 5-aminopyrazoles, containing a cyclohexylmethyl- or phenylmethyl- sulfonamido group at position-3.

Alternatively, 5-aminopyrazoles **17** containing a cyclohexylmethyl- or phenylmethyl- sulfonamido group at position-3 were prepared by treating β-ketonitriles **16** with a substituted hydrazine in the presence of Et_3_N in ethanol under reflux conditions. The intermediate **16** was obtained from β-ketoester **15** on treatment with TFA, which in turn was synthesized by condensing 4-(phenylsulfonamidomethyl)cyclohexane carboxylic acid or benzoic acid **13**, respectively, with *tert*-butyl cyanoacetate (**14**), as illustrated in Scheme 5 [36].

**Scheme 6:**
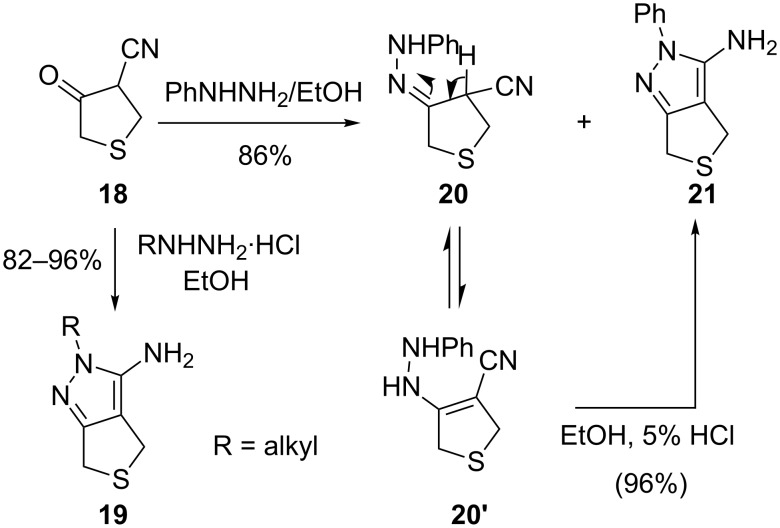
Regioselective synthesis of 3-amino-2-alkyl (or aryl) thieno[3,4-*c*]pyrazoles **19**.

Baraldi et al. [37] utilized this method for the regioselective synthesis of 2-alkyl- or 2-aryl-3-aminothieno[3,4-*c*]pyrazoles **19**. Several alkyl- or arylhydrazine hydrochlorides on condensation with 4-cyano-3-oxotetrahydrothiophene (**18**) in refluxing ethanol gave the thienopyrazoles in excellent yields. The regioselectivity of this process has been confirmed by the treatment of **18** with phenylhydrazine, which generated a mixture of intermediate hydrazone **20** and 2-phenyl-3-aminothieno[3,4-*c*]pyrazole (**21**) (Scheme 6). Hydrazones **20** on treatment with 5% HCl in ethanol underwent cyclization to afford **21**.

**Scheme 7:**
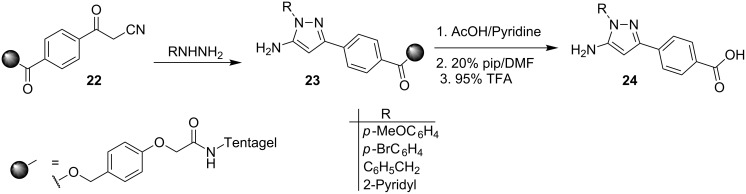
Solid supported synthesis of 5-aminopyrazoles.

A novel solid phase synthesis of some 5-aminopyrazoles **24** and their *N*-acyl and *N*-sulfonyl derivatives has recently been reported by Watson et al. [38] via the resin supported β-ketonitriles **22** (Scheme 7). The resin supported aminopyrazoles **23** were hydrolysed to yield **24** in excellent yields. The synthesis is versatile and affords compoundswith a known pharmacophoric template ideally suited for combinatorial library generation.

**Scheme 8:**
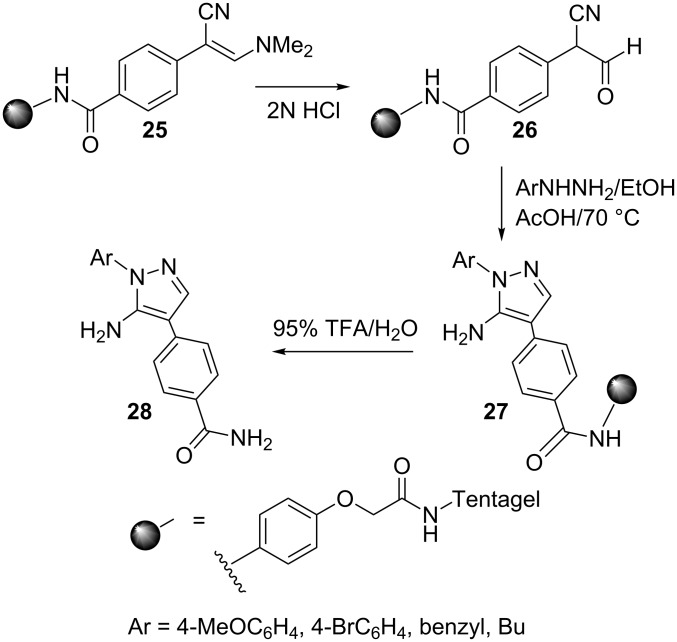
Synthesis of 5-aminopyrazoles from resin supported enamine nitrile **25** as the starting material.

Another solid phase synthesis of 5-aminopyrazoles has been reported [39] by utilizing enamine nitrile **25** as the starting material (Scheme 8). In this reaction, compound **25** was readily hydrolyzed to afford the β-ketonitrile derivative, i.e., 4-(1-cyano-2-oxoethyl)benzamide **26** which reacted efficiently with hydrazines to give the corresponding 5-aminopyrazoles **27**. Subsequent cleavage from the resin afforded 5-aminopyrazoles **28**. This new 5-aminopyrazole synthesis is more versatile and efficient than its predecessor as it avoids the use of troublesome β-ketonitrile functionality. This new route is also ideally suited for the synthesis of combinatorial libraries for drug target screening.

**Scheme 9:**
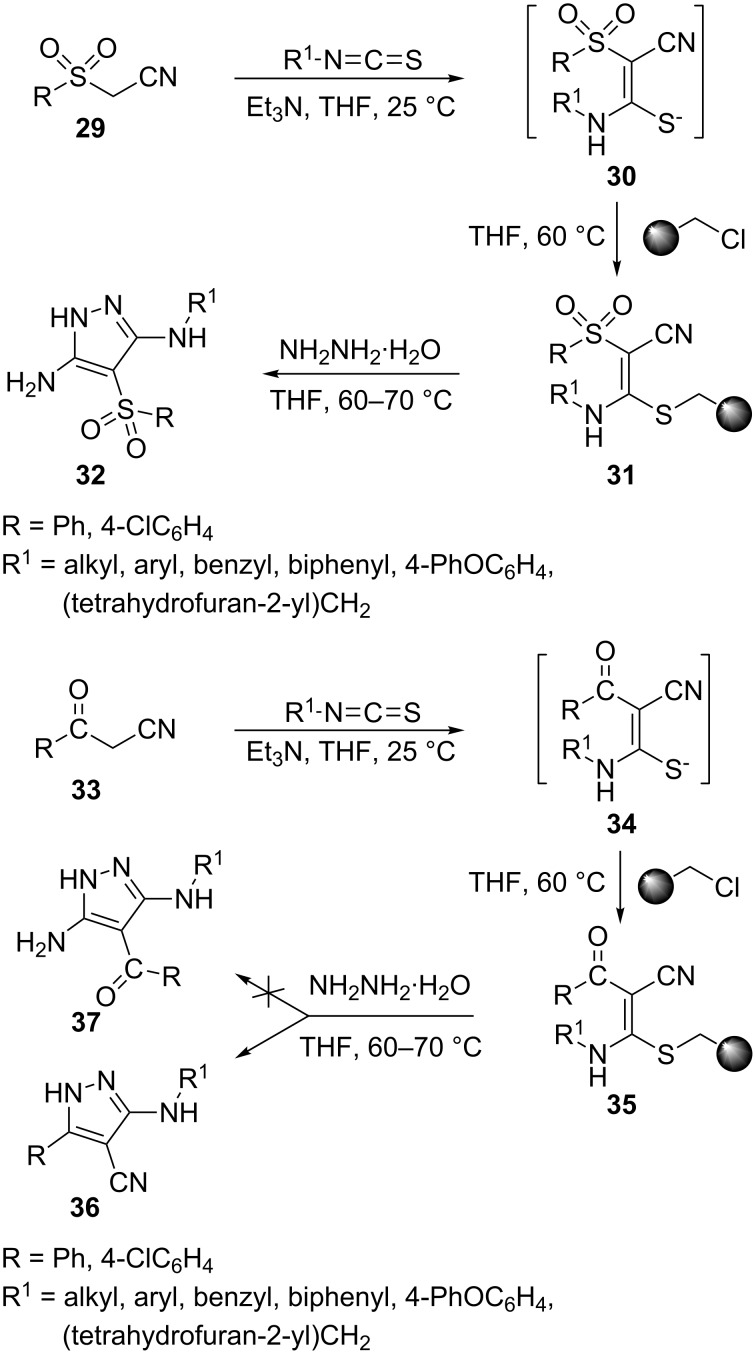
Two-step “catch and release” solid-phase synthesis of 3,4,5-trisubstituted pyrazoles.

In 2009, an efficient three-component, two-step “catch and release” solid-phase synthesis of 3,4,5-trisubstituted pyrazoles was reported which involved a base-promoted condensation of a 2-sulfonyl- or a 2-carbonyl-acetonitrile derivative (**29** or **33**) with an isothiocyanate and in situ immobilization of the resulting thiolate anion (**30** or **34**) on Merrifield resin in the first step. Reaction of the resin-bound sulfonyl intermediate **31** with hydrazine, followed by release from the resin and intramolecular cyclization, afforded 4-arylsulfonyl-3,5-diamino-1*H*-pyrazoles **32**. Reaction of the resin-bound carbonyl intermediate **35** with hydrazine, on the other hand, led to 5-aryl-3-arylamino-1*H*-pyrazole-4-carbonitriles **36**, instead of the 5-aminopyrazole **37**, which can be rationalized in terms of the higher reactivity of the carbonyl group of **35** toward hydrazine compared to the cyano group (Scheme 9) [40].

**Scheme 10:**
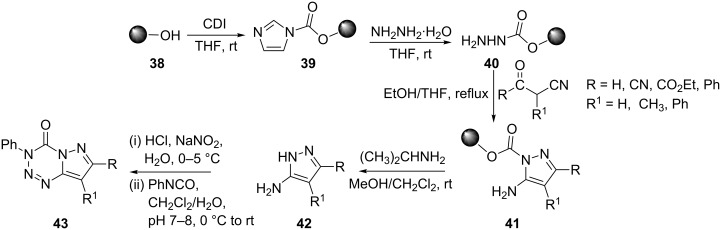
Synthesis of pyrazolo[5,1-*d*][1,2,3,5]tetrazine-4(3*H*)-ones.

Gao and Lam recently reported a solid-phase synthesis of 5-aminopyrazoles **42** which were used as precursors for the preparation of pyrazolo[5,1-*d*][1,2,3,5]tetrazine-4(3*H*)-ones **43**. Resin **39**, obtained from Wang resin **38** and a 5-10 fold excess of 1,1′-carbonyldiimidazole (CDI), was treated with hydrazine hydrate in THF at room temperature to give hydrazide resin **40**, which on further treatment with 2-(1-ethoxyethylidene)malononitrile in ethanol-CH_2_Cl_2_ (v/v 1:1) mixture at room temperature for 5 h provided resin bound 5-aminopyrazole **41**. Resin **41** was easily cleaved with isopropylamine to give crude **42**, which was diazotized with 4 M HCl and sodium nitrite in water at 0–5 °C to provide an intermediate diazonium salt. The latter underwent cycloaddition with an isocyanate in a one-pot reaction to give compound **43** (Scheme 10) [41].

**Scheme 11:**
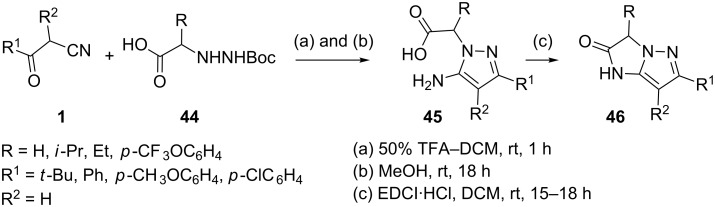
Synthesis of the 5,5-ring system, imidazo[1,2-*b*]pyrazol-2-ones.

5-Aminopyrazoles **45** have recently been prepared by Boc deprotection of the α-hydrazino acids **44** with TFA in methylene chloride followed by condensation with β-ketonitriles **1** (Scheme 11) [42]. 1-ethyl-3-[3-(dimethylamino)propyl] carbodiimide hydrochloride (EDCI) mediated intramolecular cyclodehydration resulted in the formation of the 5,5-ring system, imidazo[1,2-*b*]pyrazol-2-one **46**.

**Scheme 12:**
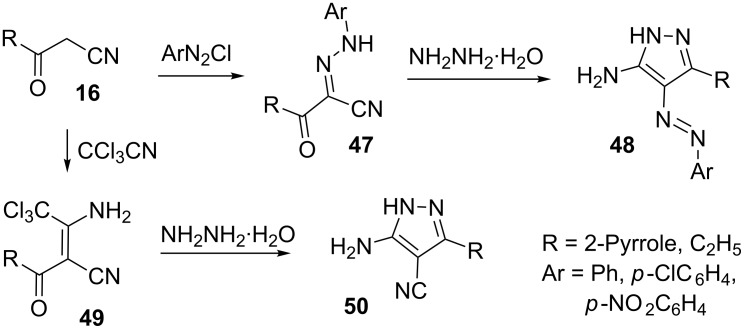
Synthesis of 5-amino-3-(pyrrol-2-yl)pyrazole-4-carbonitrile.

3-Oxopropanenitriles **16** on coupling with aromatic diazonium salts gave the corresponding 2-arylhydrazones **47**, which on treatment with hydrazine hydrate formed the 5-amino-4-arylazopyrazoles **48**. 3-Oxo-3-(pyrrol-2-yl)propanenitrile (**16**) reacted with trichloroacetonitrile to yield enamine **49**, which on further treatment with hydrazine hydrate afforded 5-amino-3-(pyrrol-2-yl)pyrazole-4-carbonitrile (**50**) (Scheme 12) [43].

**Scheme 13:**
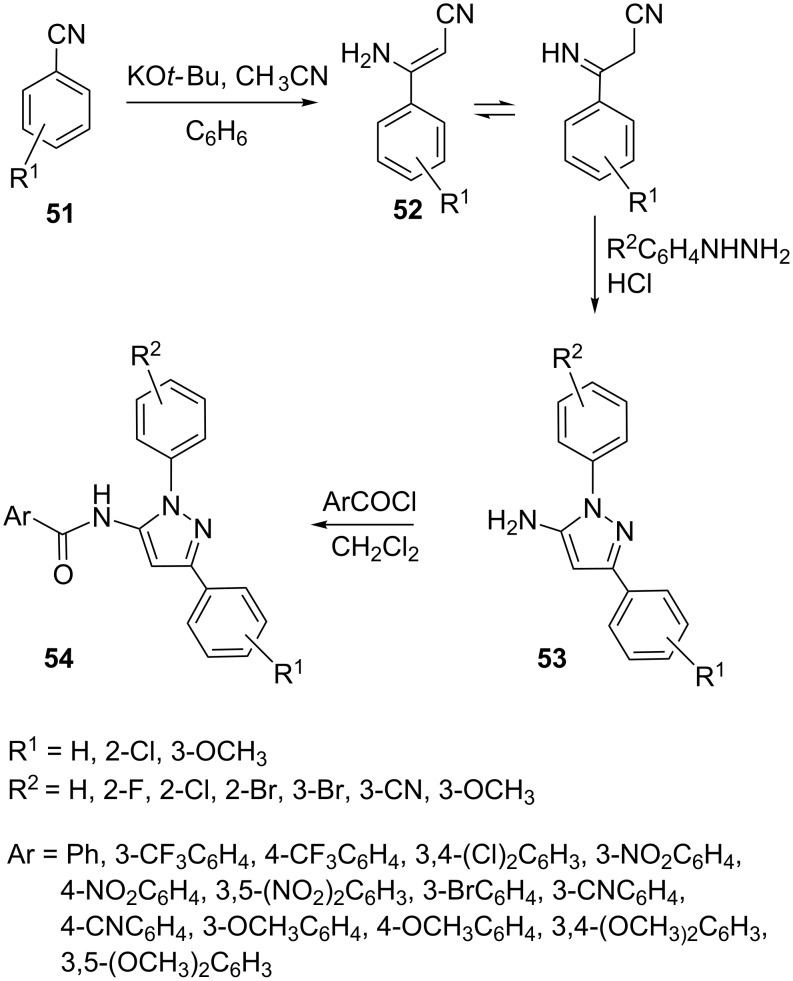
Synthesis of *N*-(1,3-diaryl-1*H*-pyrazol-5-yl)benzamide.

Synthesis of 5-amino-3-aryl-1*H*-pyrazoles **53** has been reported using benzoylacetonitrile **51** as starting material. Substituted phenylhydrazines on reaction with substituted 1-aminocinnamonitriles **52**, obtained from base catalyzed reaction of benzoylacetonitrile **51** and acetonitrile, yielded 5-amino-3-aryl-1*H*-pyrazoles **53**. Corresponding amide derivatives, i.e., *N*-(1,3-diaryl-1*H*-pyrazol-5-yl)benzamides **54** were prepared by further treating aminopyrazoles **53** with substituted benzoyl chlorides in DCM (Scheme 13) [44].

**Scheme 14:**
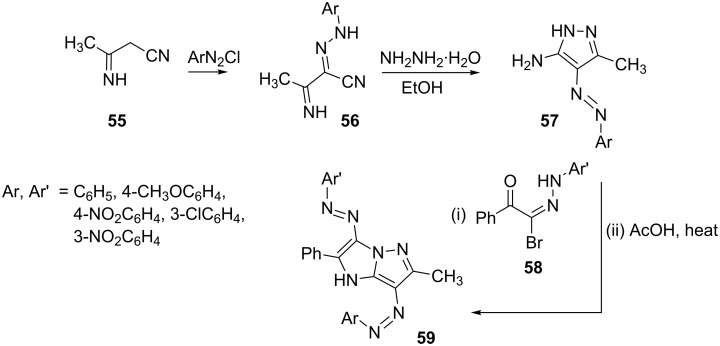
Synthesis of 3,7-bis(arylazo)-6-methyl-2-phenyl-1*H*-imidazo[1,2-*b*]pyrazoles.

3-Iminobutyronitrile (**55**) couples with aromatic diazonium salts in a similar manner to yield 2-arylhydrazono-3-iminobutyronitriles **56**. Treatment of hydrazones **56** with hydrazine hydrate in refluxing ethanol afforded the corresponding 5-amino-4-arylazo-3-methylpyrazoles **57** in good yields [45]. Pyrazoles **57** further reacted with *N*-aryl-2-oxo-2-phenylethanehydrazonoyl bromides **58** to yield 3,7-bis(arylazo)-6-methyl-2-phenyl-1*H*-imidazo[1,2-*b*]pyrazoles **59** (Scheme 14).

### 2. Reaction of malononitrile and its derivatives with hydrazines

**Scheme 15:**
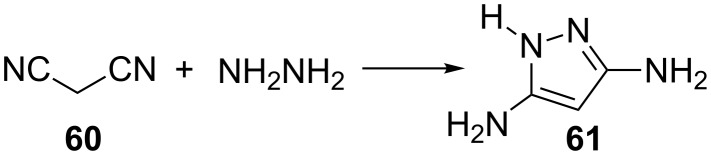
Synthesis of 3,5-diaminopyrazole.

Malononitrile (**60**) and its derivatives have been shown to react smoothly with hydrazines to yield 3,5-diaminopyrazoles that possess a wide spectrum of biological activity. As early as in 1884, Rothenburg [46] reported the simplest reaction, i.e., the condensation of malononitrile with hydrazine to give 3,5-diaminopyrazole (**61**) (Scheme 15).

**Scheme 16:**
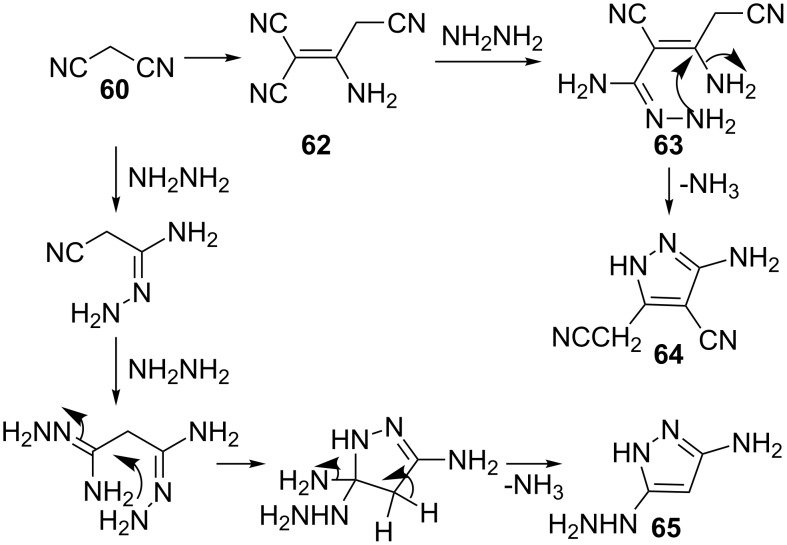
Synthesis of 5-amino-4-cyanopyrazole and 5-amino-3-hydrazinopyrazole.

The work was subsequently reinvestigated by Sato [47] who found that instead of 3,5-diaminopyrazole, two other products were produced. These compounds were characterized as 5-amino-4-cyanopyrazole **64** and 5-amino-3-hydrazinopyrazole (**65**). It was suggested that the formation of **64** resulted when two moles of malononitrile condensed with one mole of hydrazine. In this reaction dimerization of malonitrile **62** occurs before the reaction with hydrazine to give **63**. However, when one mole of malononitrile condenses with two moles of hydrazine, the formation of **65** takes place via the mechanistic pathway outlined in Scheme 16.

**Scheme 17:**
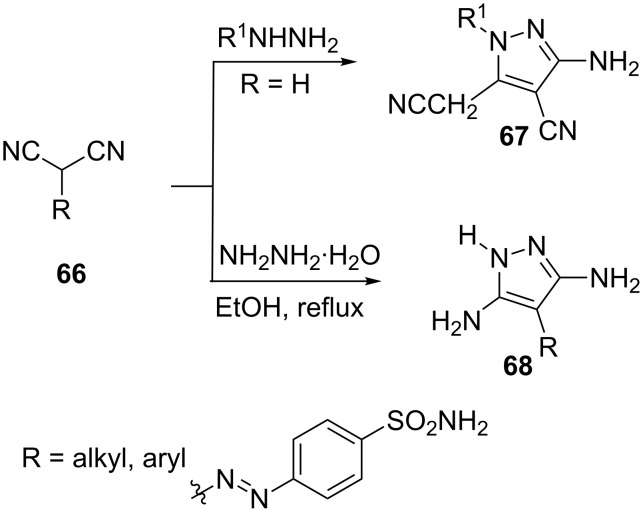
Synthesis of 3,5-diaminopyrazoles with substituted malononitriles.

The reaction of substituted hydrazines with malononitrile follows a similar course to yield **67**, [48–49] which is the 1-substituted analog of **64** (Scheme 17). However, with substituted malononitriles **66** no such dimerization is possible and the condensation with hydrazine hydrate results in the smooth formation of 3,5-diaminopyrazoles **68** (Scheme 17) [50–53].

**Scheme 18:**
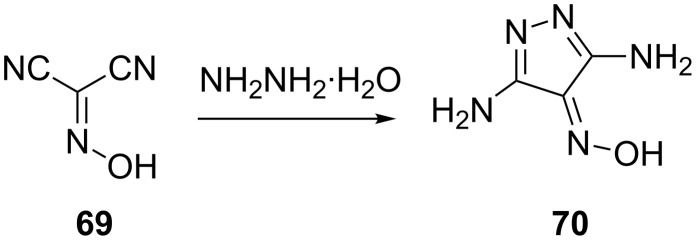
Synthesis of 3,5-diamino-4-oximinopyrazole.

Arulsamy and Bohle [54] have reported that the reaction of oximinomalononitrile (**69**) with hydrazine gives 3,5-diamino-4-oximinopyrazole (**70**) as the sole product (Scheme 18).

**Scheme 19:**
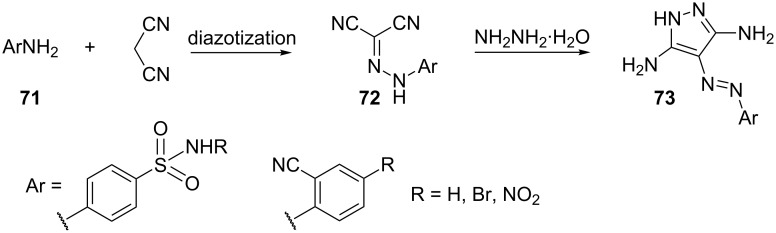
Synthesis of 4-arylazo-3,5-diaminopyrazoles.

Shvekhgeimer and Ushakova [55] have reported the synthesis of 4-arylazo-3,5-diaminopyrazoles **73** starting from substituted sulfonamides **71**. Sulfonamides **71** after diazotization undergo a coupling reaction with malononitrile to generate the hydrazones **72**, which on cycloaddition with hydrazine hydrate give the corresponding pyrazoles (Scheme 19).

Reaction of ketenes, particularly those with a cyano group at one end and a leaving group such as alkoxy, alkylthio or halogen at the other, with hydrazine and its derivatives has assumed great importance in the synthesis of 5-aminopyrazoles [56–57]. The advantage of this procedure resides in the frequent possibility of forecasting the structure of the reaction product.

**Scheme 20:**
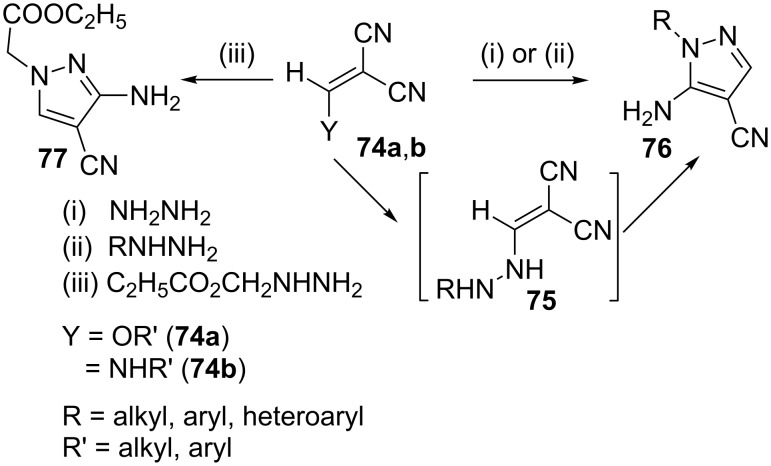
Synthesis of 3- or 5-amino-4-cyanopyrazoles.

Cheng and Robins [58] have reported the synthesis of 5-amino-4-cyanopyrazoles **76** by the reaction of hydrazines with alkoxymethylenemalononitriles **74a** (Y = OR', Scheme 20). Similar results were obtained when aminomethylenemalononitriles **74b** (Y = NHR') were treated with hydrazine indicating that reaction is initiated on the vinyl ether (vinylamine) group of **74a/b** to give 5-aminopyrazole-4-carbonitrile **76** through the intermediacy of **75** [59]. However, Elnagdi et al. [60] have reported that when ethyl hydrazinoacetate condenses with **74a** or **b**, a change in regiochemistry occurs to yield 3-amino-4-cyanopyrazoles **77** (Scheme 20).

**Scheme 21:**
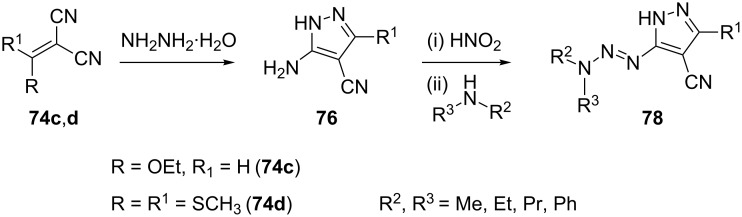
Synthesis of triazenopyrazoles.

Ethoxymethylenemalononitrile (R = OC_2_H_5_, R^1^ = H) **74c** and bis(methylthio)- methylenemalononitrile (R = R^1^ = SCH_3_) **74d** on condensation with hydrazine hydrate yield 5-aminopyrazole-4-carbonitrile **76** (R^1^ = H) and 5-amino-3-methylthiopyrazole-4-carbonitrile **76** (R^1^ = SCH_3_), respectively. These compounds were further treated with nitrous acid and coupled with different secondary amines to yield the triazenopyrazoles **78**. Compounds **78** were tested for biological activity against HIV-1 and herpes simplex viruses, and showed moderate activity against HIV-1 virus (Scheme 21) [61–62].

**Scheme 22:**
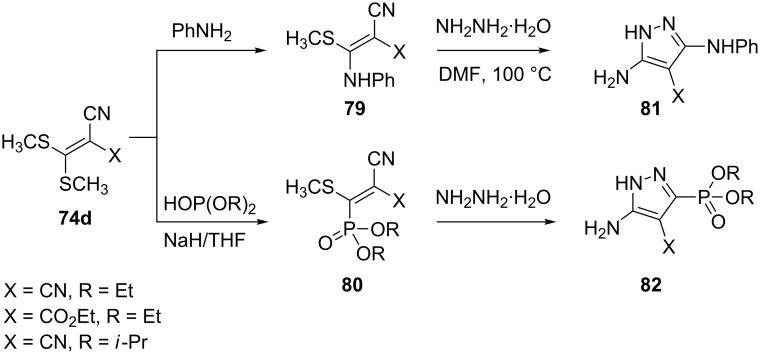
Synthesis of 5(3)-aminopyrazoles.

An interesting synthesis of 5(3)-aminopyrazoles **81** and **82** [63] has been developed using thioacetals **79** and **80** of malononitrile, which are conveniently obtained by the reaction of aniline and diethyl phosphite with bis(methylthio)methylenemalononitrile **74d**, respectively. Reaction with hydrazine monohydrate was thought to occur with loss of the methylthio group by nucleophilic attack of hydrazine and subsequent cyclization by attack on the cyano group (Scheme 22).

**Scheme 23:**
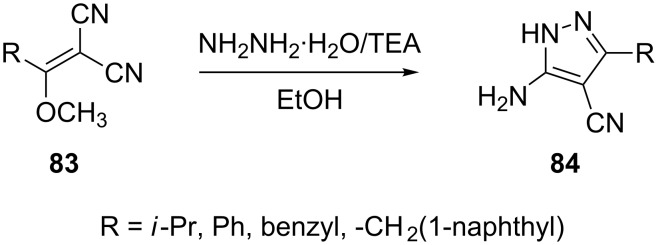
Synthesis of 3-substituted 5-amino-4-cyanopyrazoles.

The synthesis of a few 3-substituted 5-amino-4-cyanopyrazoles **84** has recently been reported by the treatment of 1,1-dicyano-2-methoxy-3-substituted propenes **83** with hydrazine hydrate in ethanolic TEA (Scheme 23) [64–65].

**Scheme 24:**
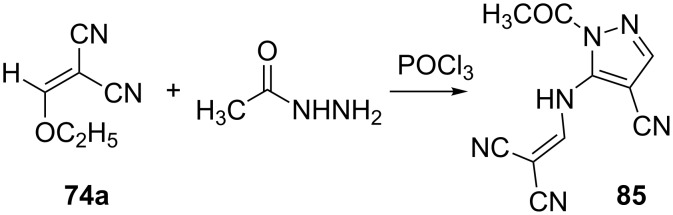
Synthesis of 2-{[(1-acetyl-4-cyano-1*H*-pyrazol-5-yl)amino]methylene}malononitrile.

Acylated hydrazine, as expected, reacts with ethoxymethylenemalononitrile **74a** in a similar manner. However, the reaction proceeds only in refluxing phosphorus oxychloride to produce compound **85** with a vinylated amino group (Scheme 24) [66].

**Scheme 25:**
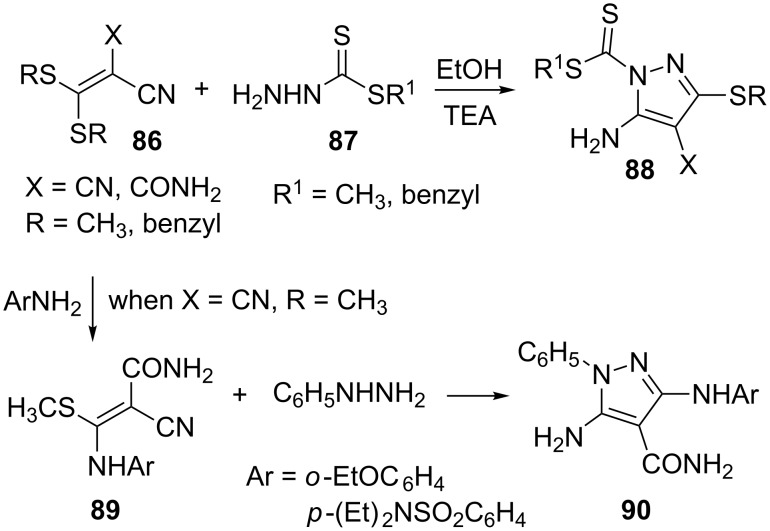
Synthesis of 5-aminopyrazole carbodithioates and 5-amino-3-arylamino-1-phenylpyrazole-4-carboxamides.

Ketene dithioacetals **86** were utilized for the synthesis of corresponding pyrazole carbodithioates **88** by cyclization with methyl- or benzylhydrazine carbodithioate **87** in ethanolic TEA at room temperature. As before, the reaction proceeds via the nucleophilic substitution of the alkylthio group by the unsubstituted nitrogen of the hydrazine. The reaction of bis(methylthio)methylenecyanoacetamide **86** (R = CH_3_, X = CONH_2_) with aromatic amines gave the corresponding 3-*N*-substituted aminoacrylamides **89**, which on further treatment with phenylhydrazine furnished the corresponding 5-amino-3-arylamino-1-phenylpyrazole-4-carboxamides **90** (Scheme 25) [67].

**Scheme 26:**
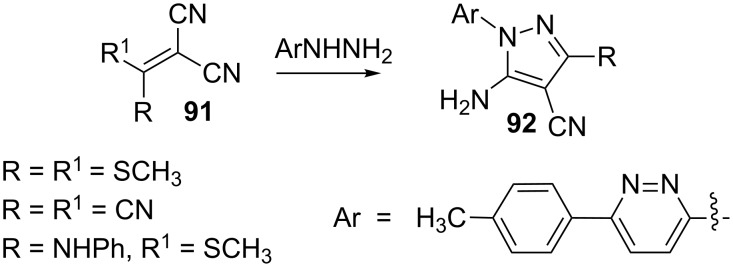
Synthesis of 5-amino-4-cyanopyrazoles.

Ketene *S*,*S*- and *S*,*N*-acetals or tetracyanoethylene **91** on reaction with 3-hydrazino-6-(*p*-tolyl)pyridazine afforded the 5-amino-4-cyanopyrazoles **92** (Scheme 26) [68].

**Scheme 27:**
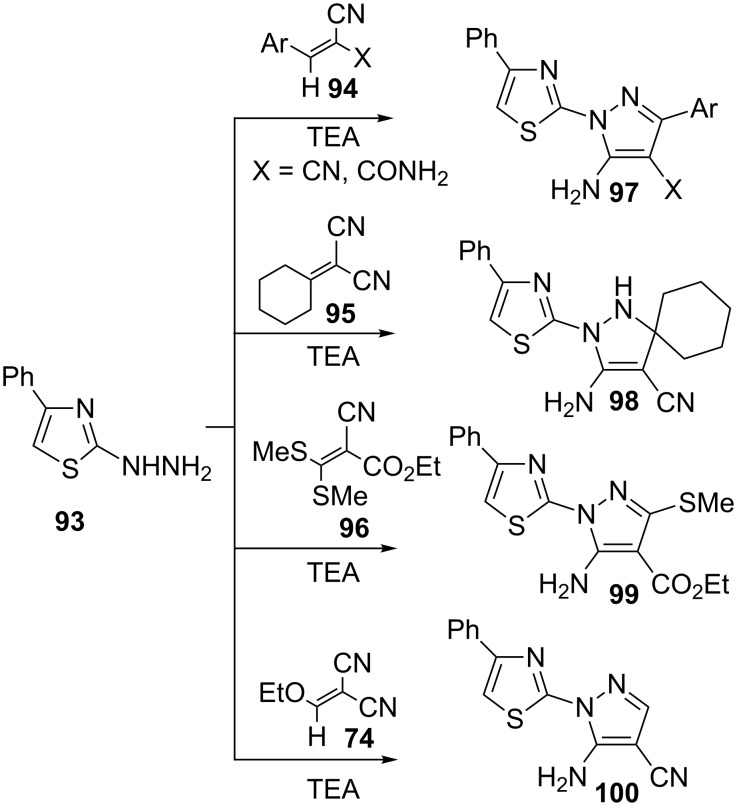
Synthesis of thiazolylpyrazoles.

Several thiazolylpyrazoles **97**–**100** bearing a variety of substituents at positions 3 and 4 were prepared by the condensation of 2-hydrazino-4-phenylthiazole (**93**) in presence of TEA with arylidenenitriles **94**, cyclohexylidene malononitrile (**95**), ethyl dimethylthiomethylene cyanoacetate (**96**) and ethoxymethylenemalononitrile (**74**), respectively, (Scheme 27) [69].

**Scheme 28:**
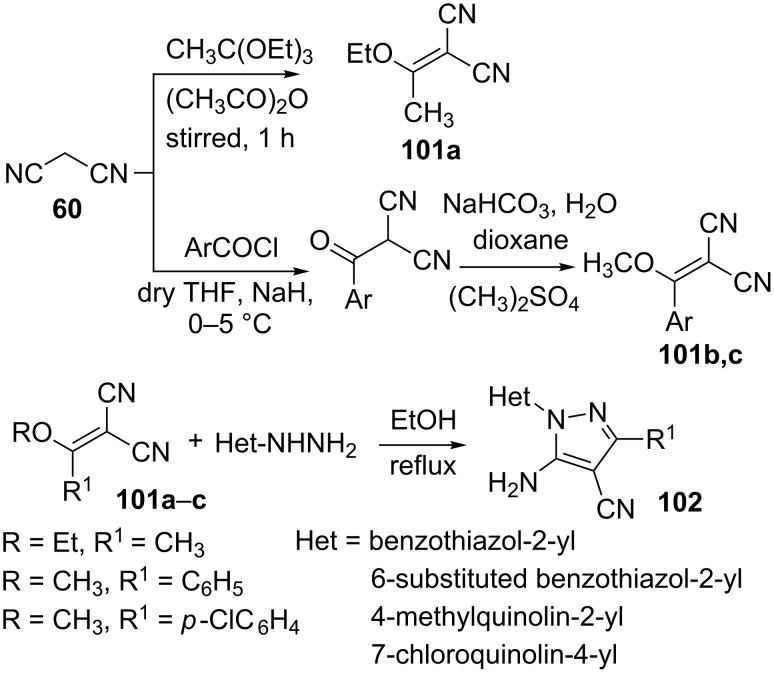
Synthesis of 5-amino-1-heteroaryl-3-methyl/aryl-4-cyanopyrazoles.

Synthesis of 5-amino-1-heteroaryl-3-methyl/aryl-4-cyanopyrazoles **102** has been carried out by us by treating various heteroarylhydrazines with alkylidenemalononitriles **101** in refluxing ethanol (Scheme 28) [70]. The starting material **101a** (R = C_2_H_5_, R^1^ = CH_3_) was obtained by the reaction of malononitrile with triethyl orthoacetate in acetic anhydride whilst methoxyarylmethylidenemalonitriles **101b,c** were obtained via a two step procedure involving the aroylation of the malonitrile with aroyl chlorides in the presence of NaH, followed by the treatment of the resulting intermediate with dimethyl sulfate.

**Scheme 29:**
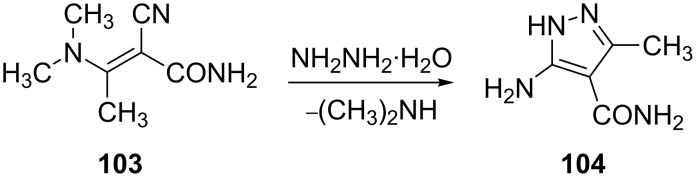
Synthesis of 5-amino-3-methylpyrazole-4-carboxamide.

Nilov et al. [71] have reported that the reaction of α-cyano-β-dimethylaminocrotonamide (**103**) with hydrazine hydrate yields 5-amino-3-methylpyrazole-4-carboxamide (**104**). The reaction proceeds by loss of dimethylamine in first step followed by cyclization via nucleophilic attack on cyano group (Scheme 29).

### 3. Miscellaneous

In addition to methods involving the reaction of hydrazine with β-ketonitriles, malononitrile and its derivatives, a number of other procedures have also been developed for the synthesis of 5-aminopyrazoles. These methods are summarized below.

**Scheme 30:**
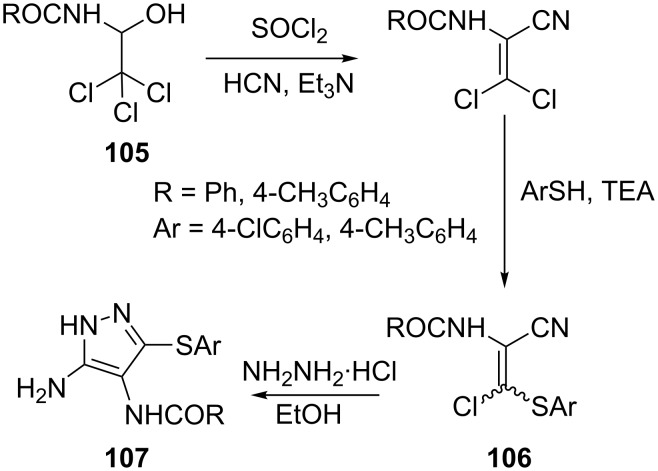
Synthesis of 4-acylamino-3(5)-amino-5(3)-arylsulfanylpyrazoles.

Synthesis of 4-acylamino-3(5)-amino-5(3)-arylsulfanylpyrazoles **107** by the reaction of 2-acylamino-3-arylsulfanyl-3-chloroacrylonitriles **106** with hydrazine hydrate has been described. Compounds **106** were readily obtained from **105**, the addition products of carboxylic acid amides and trichloroacetaldehyde, by the reaction sequence shown in the Scheme 30 [72].

**Scheme 31:**
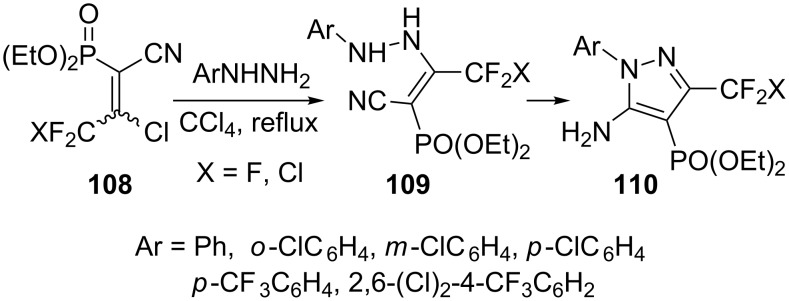
Synthesis of 5-amino-1-aryl-4-diethoxyphosphoryl-3-halomethylpyrazoles.

The reaction of 2-chloro-2-chlorodifluoro/trifluoromethyl-1-cyano-1-diethoxy phosphorylethylene **108** with arylhydrazines in refluxing carbon tetrachloride results in the rapid replacement of the chlorine atom with the terminal NH_2_ group of arylhydrazines to give intermediates **109**, which is slowly transformed into 5-amino-1-aryl-4-diethoxyphosphoryl-3-halomethylpyrazoles **110**. 2,6-Dichloro-4-trifluoromethylphenylhydrazine undergoes this reaction under more drastic conditions, i.e., prolonged refluxing (16–20 h) in carbon tetrachloride (Scheme 31) [73].

**Scheme 32:**
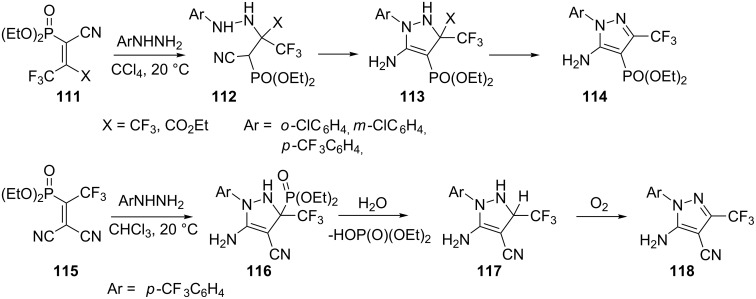
Synthesis of substituted 5-amino-3-trifluoromethylpyrazoles **114** and **118**.

Heterocyclization reactions of trifluoromethylcyanovinyl phosphonates (TFMCPs) **111** with arylhydrazines have been studied: TFMCPs **111** can be used as precursors of 2,3-dihydro-1*H*-pyrazoles **114** modified by both trifluoromethyl and diethoxyphosphoryl groups. Arylhydrazines add rapidly to the alkene double bond of **111** (X = CF_3_) at room temperature to produce an adduct which slowly cyclizes to afford 2,3-dihydro-1*H*-pyrazoles **113** in good yields. 4-Trifluoromethylphenylhydrazine also adds to ethylene **111** (X = CO_2_Et), however, the resulting adduct **112** is formed primarily as a single diastereomer and does not undergo intramolecular cyclization to pyrazoline **113** even in refluxing benzene. Further, the reaction of isomeric alkene **115** with an arylhydrazine initially forms the unstable pyrazoline **116** that transforms into pyrazole **118**. Firstly, the C–P bond apparently undergoes hydrolysis and the resulting **117** is slowly oxidized by atmospheric oxygen to yield pyrazole **118** (Scheme 32) [74].

**Scheme 33:**
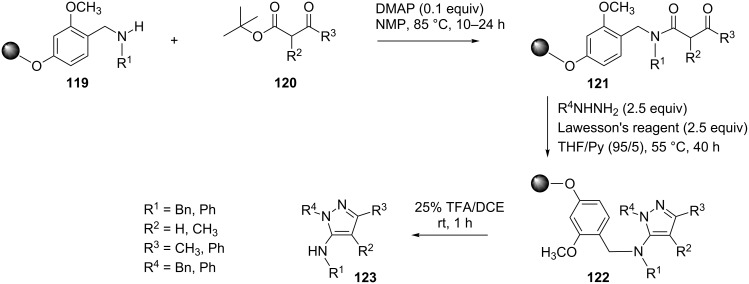
Solid-support synthesis of 5-*N*-alkylamino and 5-*N*-arylaminopyrazoles.

Dodd et al. [75] have reported an efficient solid-support synthesis of 5-*N*-alkylamino and 5-*N*-arylaminopyrazoles **123**. Heating the β-ketoesters **120** with resin-bound amines **119** in resin-compatible solvents, such as NMP or toluene, in the presence of DMAP gave the corresponding resin-immobilized β-ketoamides **121**. The latter β-ketoamides **121**, aryl- or alkylhydrazines and Lawesson’s reagent were suspended in a mixture of THF/Py and heated at 50–55 °C to afford resin-bound 5-aminopyrazoles **122**. The free 5-aminopyrazoles **123** were liberated from the solid support by treatment with TFA (Scheme 33).

**Scheme 34:**
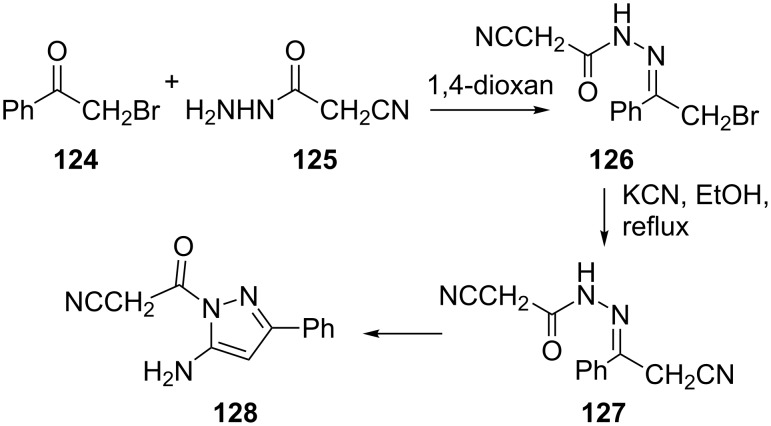
Synthesis of 5-amino-1-cyanoacetyl-3-phenyl-1*H*-pyrazole.

The reaction of cyanoacetylhydrazine (**125**) with α-bromoacetophenone (**124**) gave the *N*-[2-bromo-1-phenylethylidene]-2-cyanoacetohydrazide (**126**). Compound **126** readily underwent cyclization when treated with potassium cyanide to give 5-amino-1-cyanoacetyl-3-phenyl-1*H*-pyrazole (**128**) through the intermediacy of the acyclic cyano derivative **127** (Scheme 34) [76].

**Scheme 35:**
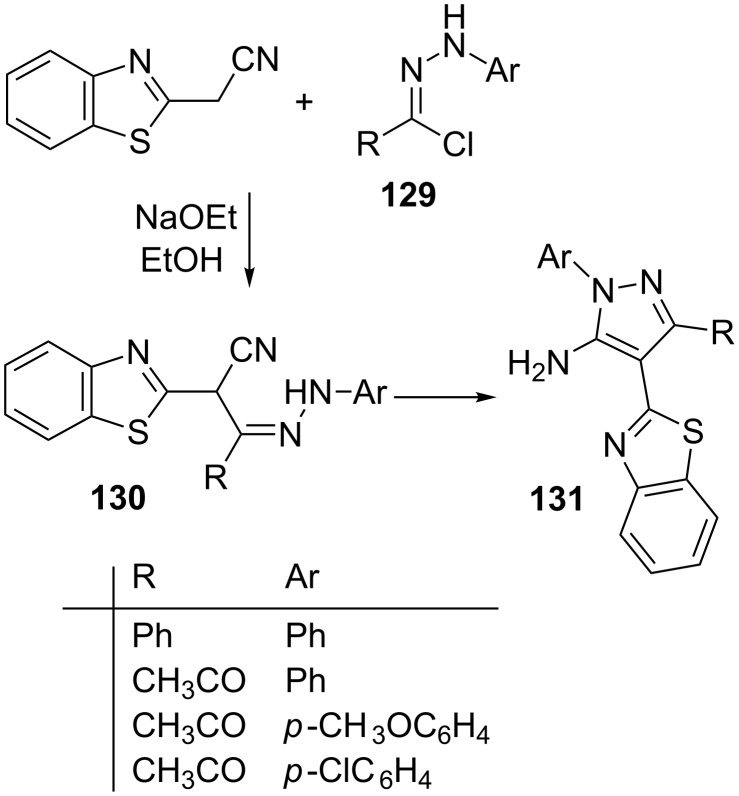
Synthesis of 3-substituted 5-amino-1-aryl-4-(benzothiazol-2-yl)pyrazoles.

Hydrazonoyl chlorides **129** on treatment with benzothiazole-2-acetonitrile in ethanolic sodium ethoxide solution at room temperature afforded intermediate hydrazones **130** which on cyclization gave products identified as 3-substituted 5-amino-1-aryl-4-(benzothiazol-2-yl)pyrazoles **131** (Scheme 35) [77].

**Scheme 36:**
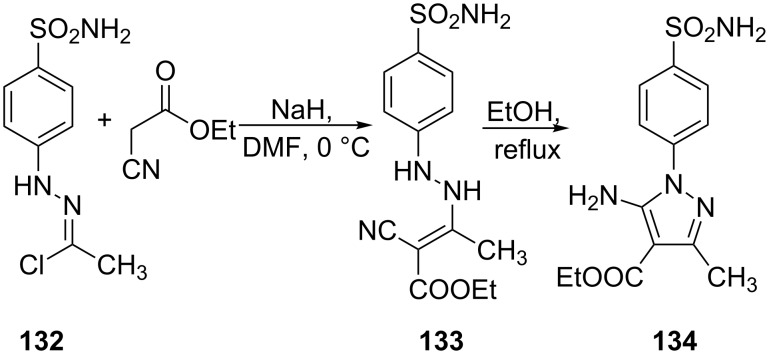
Synthesis of 5-amino-4-carbethoxy-3-methyl-1-(4-sulfamoylphenyl)pyrazole.

Similarly, hydrazonyl chloride **132** on treatment with ethyl cyanoacetate in NaH/DMF at 0 °C gave intermediate **133** which underwent cyclization to afford 5-amino-4-carbethoxy-3-methyl-1-(4-sulfamoylphenyl)pyrazole **134** (Scheme 36) [78].

**Scheme 37:**
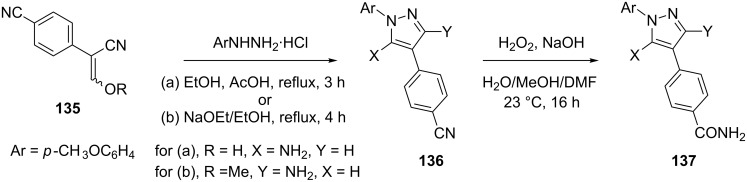
Synthesis of inhibitors of hsp27-phosphorylation and TNFa-release.

The synthetic precursor **136** for preparation of 5-aminopyrazole **137** was obtained as the major product from the acidic cyclization of the hydrazine with enol **135** (R = H). By contrast, cyclization of the hydrazine with methyl ether **135** (R = Me) under basic conditions, completely reverts the regioselectivity of this reaction and the 3-aminopyrazole intermediate **136** was obtained in excellent yield (93%) as a single isomer. The new derivatives **137** were shown to inhibit intracellular phosphorylation of hsp27 as well as LPS-induced TNFa release in cells (Scheme 37) [79].

**Scheme 38:**
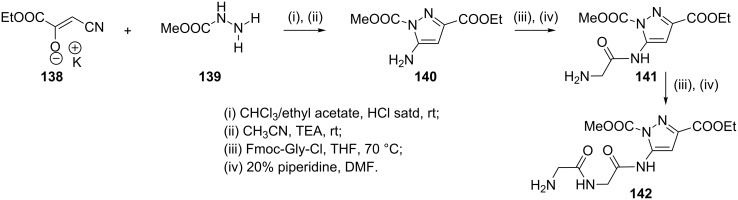
Synthesis of the diglycylpyrazole **142**.

The potassium salt of ethyl cyanopyruvate **138** on reaction with methyl carbazate **139** in a mixture of chloroform and ethyl acetate, saturated with hydrogen chloride resulted in situ protonation of the potassium salt followed by formation of intermediate hydrazone. Further treatment with TEA in acetonitrile resulted in cyclization and furnished the new 5-aminopyrazole acid ester **140**. Reaction of **140** with the acid chloride of (9-fluoroenylmethyl)carbamate(Fmoc)-protected glycine led to peptide coupling and subsequent Fmoc deprotection with piperidine gave **141**. A second coupling step can also be performed with Fmoc-protected glycine acid chloride, which affords, again after Fmoc removal, the diglycylpyrazole **142** (Scheme 38) [80].

**Scheme 39:**
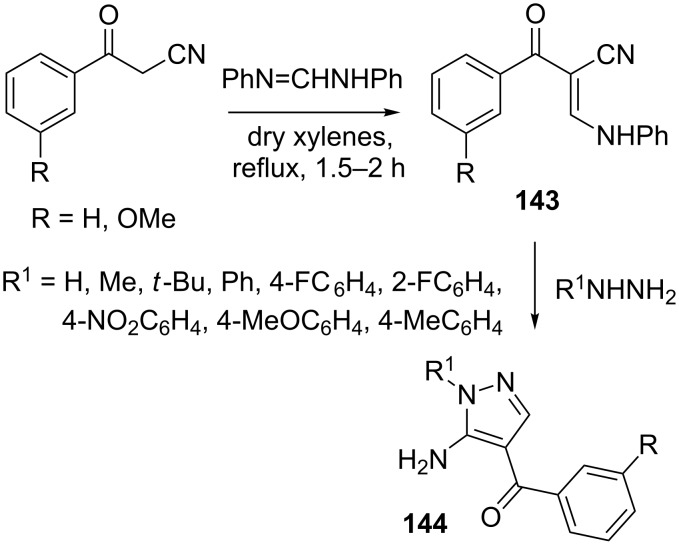
Synthesis of 5-amino-1-aryl-4-benzoylpyrazole derivatives.

A new synthetic route [81] to 5-amino-1-aryl-4-benzoyl pyrazole derivatives **144** involves the reaction of β-ketonitriles with *N*,*N*’-diphenylformamidine to give initially the cyclocondensation precursors **143** which is then transformed to **144** by reaction with hydrazines (Scheme 39).

**Scheme 40:**
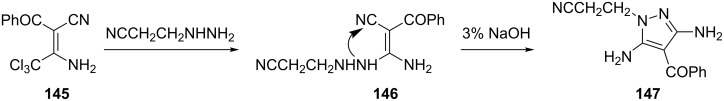
Synthesis of 4-benzoyl-3,5-diamino-1-(2-cyanoethyl)pyrazole.

The enamine nitrile **145** reacts readily with 3-hydrazinopropanenitrile to yield **146** via elimination of chloroform by the attack of less hindered nitrogen of reagent. Cyclization by treatment of the latter with 3% NaOH solution gave 4-benzoyl-3,5-diamino-1-(2-cyanoethyl)pyrazole **147** (Scheme 40) [82].

**Scheme 41:**
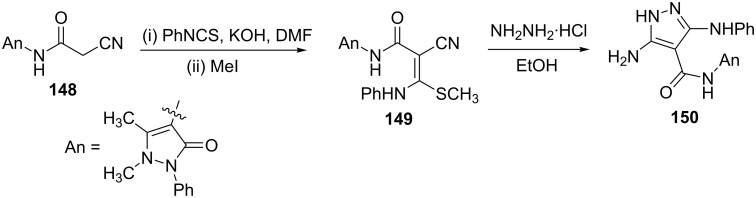
Synthesis of the 5-aminopyrazole derivative **150**.

2-Cyano-*N*-(1,5-dimethyl-3-oxo-2-phenyl-2,3-dihydro-1*H*-pyrazol-4-yl)acetamide (**148**) was utilized for the synthesis of the 5-aminopyrazole **150**. Treatment of **148** with phenyl isothiocyanate in DMF in the presence of potassium hydroxide at room temperature, followed by treatment with methyl iodide afforded the novel ketene *N*,*S*-acetal **149**. Reaction of **149** with hydrazine in refluxing ethanol gave the corresponding 5-aminopyrazole derivative **150**. The reaction proceeds in the usual manner, i.e., loss of methylthio group by nucleophilic attack of hydrazine in the first step followed by the cyclization (Scheme 41) [83].

**Scheme 42:**
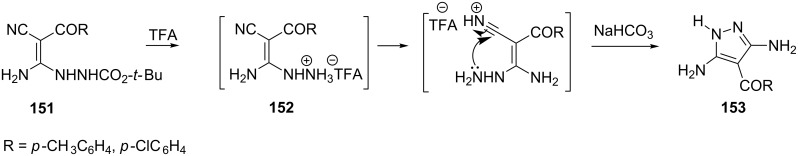
Synthesis of 3,5-diaminopyrazoles **153**.

Hutaud et al. [84] have reported a unique method for the preparation of 3,5-diaminopyrazoles **153** in good yields by the treatment of the enamine nitrile **151** with trifluoroacetic acid. Boc deprotection by trifluoroacetic acid to **152** is followed by spontaneously nucleophilic attack on the cyano group by the *N*-terminal nitrogen of the hydrazine substitutent (Scheme 42).

**Scheme 43:**
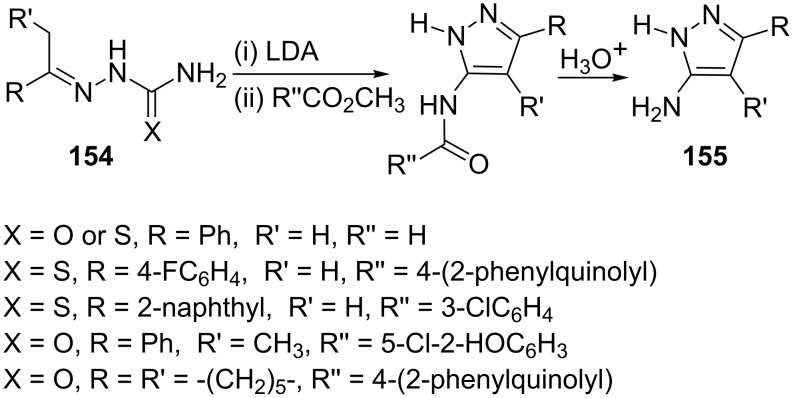
Synthesis of 5-aminopyrazoles derivatives **155** via lithiated intermediates.

Beam et al. [85] have reported a novel synthesis of 5-aminopyrazoles **155** from polylithiated *C*(α), *N*-thiosemicarbazones (X = S) or *C*(α), *N*-semicarbazones (X = O). The polylithiated intermediates, prepared from *C* (α), *N*-thiosemicarbazones (X = S) or *C* (α), *N*-semicarbazones (X = O) **154** and an excess of lithium diisopropylamide (LDA), underwent cyclization and on subsequent hydrolysis gave the 5-aminopyrazole derivatives **155** (Scheme 43).

**Scheme 44:**
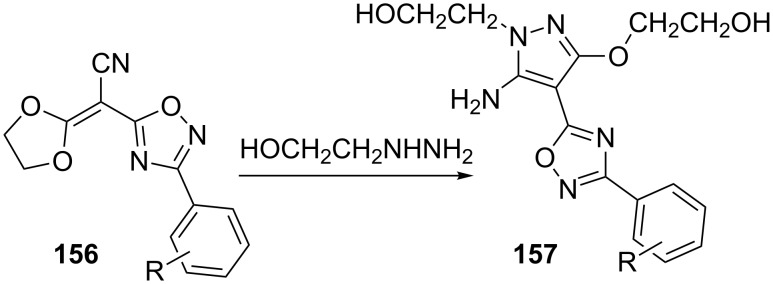
Synthesis of 5-amino-4-(1,2,4-oxadiazol-5-yl)-pyrazoles **157**.

It has been reported that 1,2,4-oxadiazolylmethylenedioxolanes **156** undergo cyclization on treatment with 2-hydroxyethylhydrazine to give 5-amino-4-(1,2,4-oxadiazol-5-yl)-pyrazoles [86] **157** (Scheme 44).

**Scheme 45:**
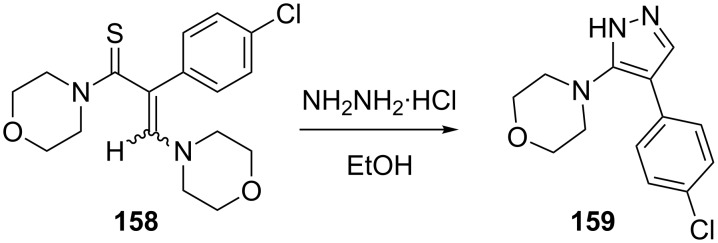
Synthesis of a 5-aminopyrazole with anticonvulsant activity.

The reaction of 3-aminothioacrylamide **158** with hydrazine hydrochloride has been reported to furnish the 5-aminopyrazole **159** in good yield. Various derivatives were tested for anticonvulsant activity in a variety of test models (Scheme 45) [87].

**Scheme 46:**
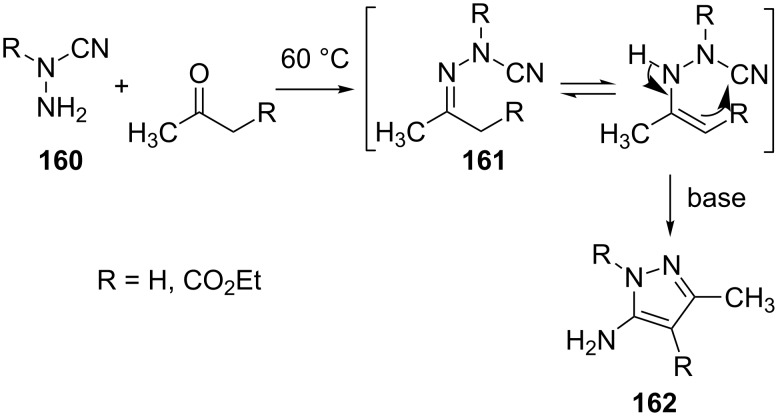
Synthesis of tetrasubstituted 5-aminopyrazole derivatives.

Another interesting synthesis that affords tetrasubstituted 5-aminopyrazole derivatives **162** involves the reaction of *N*,*N*-disubstituted hydrazines **160** with ketones [88]. The hydrazones **161** so formed undergo cyclization in the presence of base to yield the desired compounds **162** (Scheme 46).

**Scheme 47:**
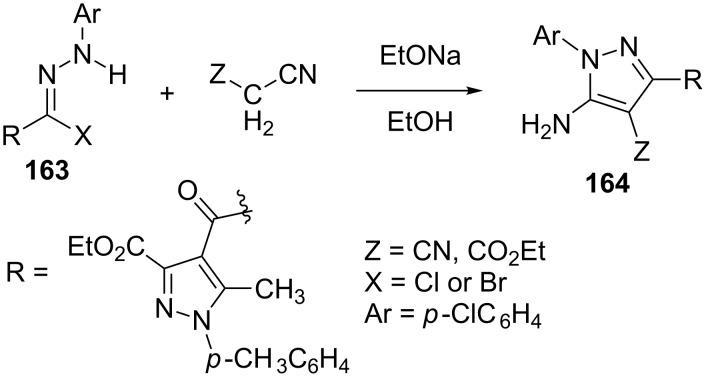
Synthesis of substituted 5-aminopyrazoles from hydrazonoyl halides.

Abdelhamid et al. [89–90] have reported the synthesis of substituted 5-aminopyrazoles **164** by the treatment of active methylene compounds such as malononitrile, ethyl cyanoacetate etc. with hydrazonoyl halides **163** in ethanolic sodium ethoxide (Scheme 47).

**Scheme 48:**
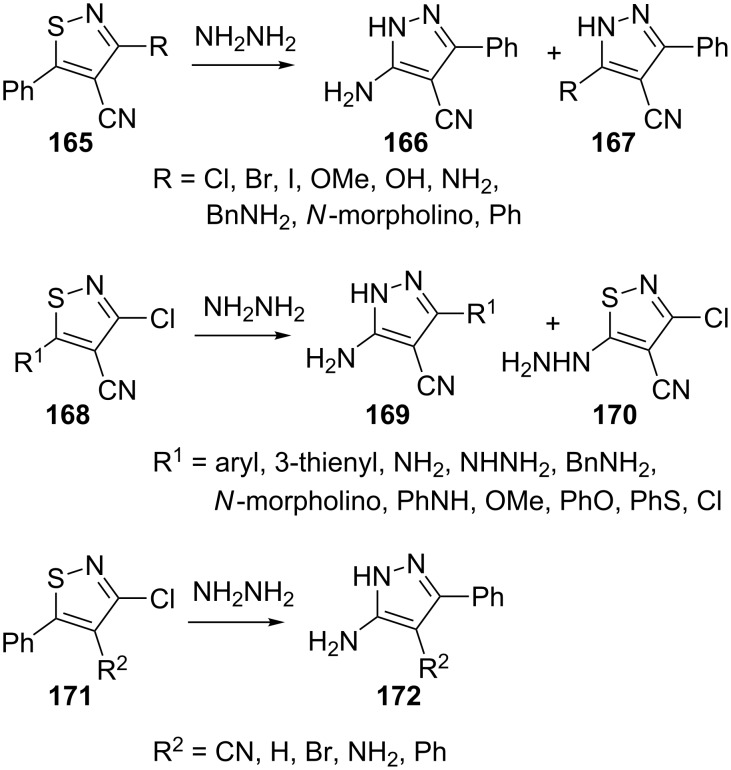
Synthesis of 3-amino-5-phenylpyrazoles from isothiazoles.

Ioannidou and Koutentis [91] investigated the conversion of isothiazoles into pyrazoles on treatment with hydrazine. The influence of various C-3, C-4 and C-5 isothiazole substituents and some limitations of this ring transformation were investigated. When a good nucleofugal group (e.g., Cl, Br and I) is present at C-3 in the isothiazole **165**, it is replaced by an amino group and 5-aminopyrazoles **166** are obtained. However, when the 3-substituent is not a good leaving group it is retained in the pyrazole product **167**. A series of 3-chloro-5-substituted isothiazole-4-carbonitriles **168** bearing steric and/or electronic constraints at C-5 were also treated with anhydrous hydrazine and the corresponding 3-aminopyrazoles **169** were obtained in varying yields. However, when the substituent at C-5 in isothiazole was a better nucleofuge (e.g., PhO, PhS and Cl), the 5-hydrazinoisothiazole **170** was rapidly produced in good yield. Several isothiazoles **171** with a variety of C-4 substituents were also reacted with anhydrous hydrazine to yield the corresponding 3-amino-5-phenylpyrazoles **172**. Reaction time and the yield of the reaction was dependent on the substituents present (Scheme 48).

**Scheme 49:**
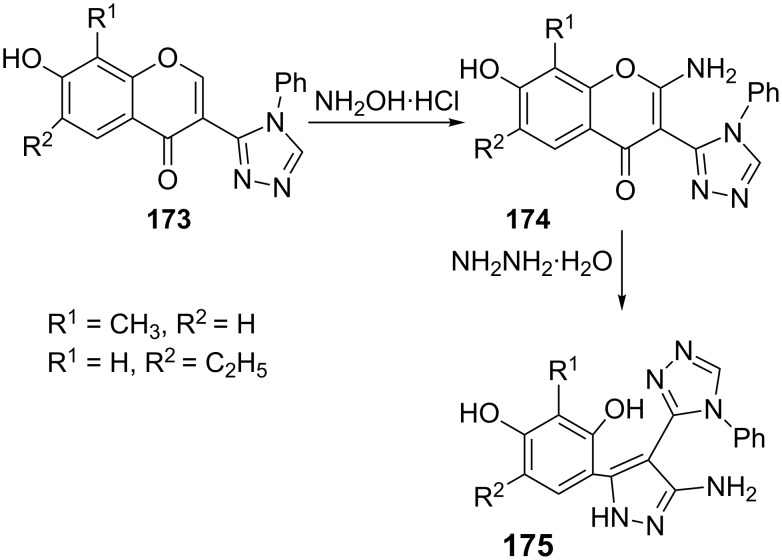
Synthesis of 5-aminopyrazoles via ring transformation.

The reaction of hydroxylamine with 3-(4-phenyl-1,2,4-triazol-3-yl)chromones **173** has been reported to give the 2-aminochromones **174**. The 2-aminochromones **174** undergo ring transformation to afford the 5-aminopyrazoles **175** but only upon prolonged heating with hydrazine hydrate in high boiling alcohols (2-propanol, butanol) or in DMF (Scheme 49) [92].

## Conclusion

5-Aminopyrazole is an important heterocyclic system which has great significance in pharmaceutical industry as well as being a useful synthon for the synthesis of many bridgehead heterocycles. This review describes new strategies and the development of novel concepts along with conventional methods to synthesize a wide variety of substituted 5-aminopyrazoles. Conventional methods such as condensation of β-ketonitriles, malononitrile and its derivatives with hydrazines in addition to modern methods of resin supported solid-phase synthesis, multi-component synthesis and ring transformations provide useful synthetic routes to 5-aminopyrazoles.

## References

[1] Elguero J, Katritzky A R, Rees C W (1984). Comprehensive Heterocyclic Chemistry.

[2] Elguero J, Katritzky A R, Rees C W, Scriven E F V (1996). Comprehensive Heterocyclic Chemistry II.

[3] Kost A N, Grandberg I I, Katritzky A R, Boulton A J (1966). Advances in Heterocyclic Chemistry.

[4] Lee K Y, Kim J M, Kim J N (2003). Tetrahedron Lett.

[5] Wiley R H, Wiley P (1964). Pyrazolones, Pyrazolidones and Derivatives.

[6] Behr L C, Fusco R, Jarboe C H, Weissberger A (1967). The Chemistry of Heterocyclic Compounds, Pyrazoles, Pyrazolines, Pyrazolidines, Indazoles and Condensed Rings.

[7] David D P, Martin D J, Charles M D F (1997). 5-Aminopyrazoles useful as selective inhibitors of the protein tyrosine kinase P56ick. WO.

[8] Kordik C P, Luo C, Zanoni B C, Lovenberg T W, Wilson S J, Vaidya A H, Crooke J J, Rosenthal D I, Reitz A B (2001). Bioorg Med Chem Lett.

[9] Nakazato A, Okuyama S (1999). Drugs Future.

[10] Meegalla S K, Doller D, Sha D, Soll R, Wisnewski N, Silver G M, Dhanoa D (2004). Bioorg Med Chem Lett.

[11] Huppatz J L (1985). Aust J Chem.

[12] Shamroukh A H, Rashad A E, Sayed H H (2005). Phosphorus, Sulfur Silicon Relat Elem.

[13] Carter T A, Wodicka L M, Shah N P, Velasco A M, Fabian M A, Treiber D K, Milanov Z V, Atteridge C E, Biggs W H, Edeen P T (2005). Proc Natl Acad Sci U S A.

[14] An H, Eum S-J, Koh M, Lee S K, Park S B (2008). J Org Chem.

[15] Abu Elmaati T M, El-Taweel F M (2004). J Heterocycl Chem.

[16] Selleri S, Gratteri P, Costagli C, Bonaccini C, Costanzo A, Melani F, Guerrini G, Ciciani G, Costa B, Spinetti F (2005). Bioorg Med Chem.

[17] Gopalsamy A, Yang H, Ellingboe J W, Tsou H-R, Zhang N, Honores E, Powell D, Miranda M, McGinnis J P, Rabindran S K (2005). Bioorg Med Chem Lett.

[18] Zhang X-Y, Li X-Y, Fan X-S, Wang X, Qu G-R, Wang J-J (2009). Heterocycles.

[19] Elnagdi M H, Elgemeie G E H, Abd-Elaal F A-E (1985). Heterocycles.

[20] Bagley M C, Davis T, Dix M C, Widdowson C S, Kipling D (2006). Org Biomol Chem.

[21] Butler D E, Alexander S M (1982). J Heterocycl Chem.

[22] Elnagdi M H, Elfahham H A, Elgemeie G E H (1983). Heterocycles.

[23] Elnagdi M H, Elmoghayar M R H, Elgemeie G E H (1984). Synthesis.

[24] Sunder S, Peet N P (1980). J Heterocycl Chem.

[25] Elderfield R C (1957). Heterocyclic Compounds.

[26] Snyder H R (1975). J Heterocycl Chem.

[27] Senga K, Robins R K, Brien D E (1975). J Heterocycl Chem.

[28] Joshi K C, Pathak V N, Garg U (1979). J Heterocycl Chem.

[29] Singh S P, Prakash O, Tomar R K, Sawhney S N (1978). Indian J Chem.

[30] Singh S P, Tomar R K, Prakash O, Sawhney S N (1979). Indian J Chem.

[31] Smith P A S, Ahmad Y (1971). J Org Chem.

[32] Kumar V, Aggarwal R, Tyagi P, Singh S P (2005). Eur J Med Chem.

[33] Sosnovskikh V Y, Sizon A Y, Usachev B I (2002). Russ Chem Bull.

[34] Aggarwal R, Kumar V, Tyagi P, Singh S P (2005). Bioorg Med Chem.

[35] Jachak M, Krießmann U, Mittelbach M, Junek H (1993). Monatsh Chem.

[36] Kordik C P, Luo C, Zanoni B C, Dax S L, McNally J J, Lovenberg T W, Wilson S J, Reitz A B (2001). Bioorg Med Chem Lett.

[37] Baraldi P G, El-Kashef H, Manfredini S, de las Infantas M J P, Romagnoli R, Spalluto G (1998). Synthesis.

[38] Watson S P, Wilson R D, Judd D B, Richards S A (1997). Tetrahedron Lett.

[39] Wilson R D, Watson S P, Richards S A (1998). Tetrahedron Lett.

[40] Ma W, Peterson B, Kelson A, Laborde E (2009). J Comb Chem.

[41] Gao Y, Lam Y (2010). J Comb Chem.

[42] Blass B E, Srivastava A, Coburn K R, Faulkner A L, Janusz J J, Ridgeway J M, Seibel W L (2004). Tetrahedron Lett.

[43] Hassaneen H M E (2007). Synth Commun.

[44] de Paulis T, Hemstapat K, Chen Y, Zhang Y, Saleh S, Alagille D, Baldwin R M, Tamagnan G D, Conn P J (2006). J Med Chem.

[45] Shawali A S, Mosselhi M A, Altablawy F M A, Farghaly T A, Tawfik N M (2008). Tetrahedron.

[46] Rothenburg E (1894). Chem Ber.

[47] Sato T (1959). J Org Chem.

[48] Grey E J, Stevens H N E, Stevens M P G (1978). J Chem Soc, Perkin Trans 1.

[49] Taylor E C, Hartke K S (1959). J Am Chem Soc.

[50] Echevarría A, Martín M, Pérez C, Rozas I (1994). Arch Pharm.

[51] Vaquero J J, Fuentes L, Del Castillo J C, Pérez M I, García J L, Soto J L (1987). Synthesis.

[52] Hassanien A A, Amar A E, Ghozlan S A S (2000). J Chin Chem Soc.

[53] Elnagdi M H, Abd Allah S O (1973). J Prakt Chem.

[54] Arulsamy N, Bohle D (2000). J Org Chem.

[55] Shvekhgeimer M-G A, Ushakova O A (2001). Chem Heterocycl Compd.

[56] Cankar P, Wiedermannova I, Slouka J (2002). Acta Univ Palacki Olomuc, Fac Rerum Nat, Chem.

[57] Dyachenko V D, Tkachev R P (2006). Russ J Org Chem.

[58] Cheng C C, Robins R K (1956). J Org Chem.

[59] Dooley M J, Quinn R J, Scammells P J (1989). Aust J Chem.

[60] Elnagdi M H, Hafez E A A, El-Fahham H A, Kandeel E M (1980). J Heterocycl Chem.

[61] Larsen J S, Zahran M A, Pedersen E B, Nielsen C (1999). Monatsh Chem.

[62] Howe R K, Bolluyt S C (1969). J Org Chem.

[63] Lu R-J, Yang H-Z (1997). Tetrahedron Lett.

[64] Kraybill B C, Elkin L L, Blethrow J D, Morgan D O, Shokat K M (2002). J Am Chem Soc.

[65] Kluge R, Schulz M, Pobišova M, Nüchter M (1994). Chem Ber.

[66] Quinn R J, Scammells P J, Kennard C H L, Smith G (1991). Aust J Chem.

[67] Hassan S M, Emam H A, Abdelall M M (2001). Phosphorus, Sulfur Silicon Relat Elem.

[68] Al-Afaleq E I, Abubshait S A (2001). Molecules.

[69] Amer A A (2008). Phosphorus, Sulfur Silicon Relat Elem.

[70] Aggarwal R, Kumar V, Singh S P (2006). Indian J Chem.

[71] Nilov D B, Solov’eva N P, Nikolaeva I S, Peters V V, Krylova L Y, Gus’kova T A, Granik V G (1998). Pharm Chem J.

[72] Popil’nichenko S V, Pil’o S G, Brovarets G B S, Chernega A N, Drach B S (2005). Russ J Gen Chem.

[73] Shidlovskii A F, Peregudov A S, Averkiev B B, Antipin M Y, Chkanikov N D (2004). Russ Chem Bull.

[74] Shidlovskii A F, Peregudov A S, Bulychev Y N, Chkanikov N D (2009). Pharm Chem J.

[75] Dodd D S, Martinez R L, Kamau M, Ruan Z, Van Kirk K, Cooper C B, Hermsmeier M A, Traeger S C, Poss M A (2005). J Comb Chem.

[76] Wardakhan W W, Louca N A (2007). J Chil Chem Soc.

[77] Dawood K M (1998). J Chem Res, Synop.

[78] Organ M G, Mayer S (2003). J Comb Chem.

[79] Velcicky J, Feifel R, Hawtin S, Heng R, Huppertz C, Koch G, Kroemer M, Moebitz H, Revesz L, Scheufler C (2010). Bioorg Med Chem Lett.

[80] Rzepecki P, Gallmeier H, Geib N, Cernovska K, König B, Schrader T (2004). J Org Chem.

[81] Bagley M C, Davis T, Dix M C, Murziani P G S, Rokicki M J, Kipling D (2008). Bioorg Med Chem Lett.

[82] Elmoghayar M R H, Elghandour A H H (1986). Monatsh Chem.

[83] Bondock S, Rabie R, Etman H A, Fadda A A (2008). Eur J Med Chem.

[84] Hutaud D H, Baudy-Floc’h M, Gougeon P, Gall P, Le Grel P (2001). Synthesis.

[85] Beam C F, Davis S E, Cordray T L, Chan K W, Kassis C M, Freeman-Davis J G, Latham G M, Guion T S, Hilderbran K C, Church A C (1997). J Heterocycl Chem.

[86] Neidlein R, Li S (1996). J Heterocycl Chem.

[87] Unverferth K, Engel J, Höfgen N, Rostock A, Günther R, Lankau H-J, Menzer M, Rolfs A, Liebscher J, Müller B (1998). J Med Chem.

[88] Fouqué D, About-Jaudet E, Collingnon N (1995). Synth Commun.

[89] Abdelhamid A O, Zohdi H F, Sallam M M, Ahmed N A (2000). Molecules.

[90] Abdelhamid A O (1993). J Chem Res, Synop.

[91] Ioannidou H A, Koutentis P A (2009). Tetrahedron.

[92] Shokol T V, Turov V A, Semeniuchenko V V, Krivokhizha N V, Khilya V P (2006). Chem Heterocycl Compd.

